# Non-iterative image reconstruction from sparse magnetic resonance imaging radial data without priors

**DOI:** 10.1186/s42492-020-00044-y

**Published:** 2020-04-23

**Authors:** Gengsheng L. Zeng, Edward V. DiBella

**Affiliations:** 1 Department of Radiology and Imaging Sciences, University of Utah, 729 Arapeen Drive, Salt Lake City, UT 84108 USA; 2 Department of Engineering, Utah Valley University, 800 West University Parkway, Orem, 84058 USA

**Keywords:** Tomographic image reconstruction, Under-sampled measurements, Fast magnetic resonance imaging, Analytics reconstruction

## Abstract

The state-of-the-art approaches for image reconstruction using under-sampled k-space data are compressed sensing based. They are iterative algorithms that optimize objective functions with spatial and/or temporal constraints. This paper proposes a non-iterative algorithm to estimate the un-measured data and then to reconstruct the image with the efficient filtered backprojection algorithm. The feasibility of the proposed method is demonstrated with a patient magnetic resonance imaging study. The proposed method is also compared with the state-of-the-art iterative compressed-sensing image reconstruction method using the total-variation optimization norm.

## Introduction

This paper considers image reconstruction for under-sampled magnetic resonance imaging (MRI) data, which is a typical case for fast imaging such as dynamic imaging and real-time imaging [1, 2]. Since the data is incomplete, direct image reconstruction contains severe artifacts. The state-of-the-art approaches are compressed sensing based iterative reconstruction methods. The iterative methods optimize an objective function that contains spatial and/or temporal constraints. Some standard compressed sensing papers suggest objective functions with an L1 norm to encourage sparseness [3–8]. The compressed sensing approaches can be considered as Bayesian methods, in which the prior information is formulated as the constraints. It is a popular approach that the non-Cartesian k-space measurements are interpolated into the Cartesian grid before reconstruction [9–11].

Recently machine learning is becoming a popular solution for applications in almost all areas. An important application of machine learning is image reconstruction with limited data [12–15]. On the surface, machine learning methods do not need any prior information about the image except for a large training set. In fact, the training data set is the prior information, and machine learning methods can also be considered as Bayesian methods.

One drawback of Bayesian methods is that if the object being imaged is quite different from the Bayesian assumptions, the reconstructed image from the Bayesian methods may not be trustworthy. The method proposed in this paper does not assume any prior information. Our method is non-iterative and efficient to implement.

Re-gridding data points may introduce errors to the image. Due to the nature of the filtered backprojection (FBP), our proposed method assumes radial sampling in the k-space, and the measurements do not get interpolated into the Cartesian grid.

Parallel MRI uses multiple receiver coils. The space-dependent properties of receiver coils can be employed to reduce under-sampling induced artifacts [16–18]. This paper considers only single-channel MRI. Parallel MRI is beyond the scope of this paper.

## Methods

### Linear interpolation causes rotated shadow images

In this paper, we only consider radial k-space sampling. Under-sampled k-space here implies that the number of views is not sufficient. In other words, the angular sampling is sparse. Typically streaking aliasing artifacts will appear in the reconstructed images if the angular sampling is not fine enough. It is noticed that the simple linear interpolation method to estimate the unmeasured measurements has never been used in under-sampled MRI applications, and in the first section of this paper, we investigate the reasons why the naïve linear interpolation approach does not work well.

Here we use a simple example in the spatial domain to illustrate our point. Let us refer to the one-dimensional (1D) inverse Fourier transform in the radial direction of the k-space measurements as the *sinogram*. Let the sinogram be *p*(*n*, *m*), where *n* is the index along the radial direction and *m* is the view angle index. When *m* is odd, *p*(*n*, *m*) is assumed to be measured. When *m* is even, *p*(*n*, *m*) is not measured and needs to be estimated. A simple linear interpolation scheme to estimate *p*(*n*, 2 *m*) from *p*(*n*, 2 *m* - 1) and *p*(*n*, 2 *m* + 1) is

1 $$ p\left(n,2m\right)=0.5\times \left[p\left(n,2m-1\right)+p\left(n,2m+1\right)\right] $$

**Fig. 1:**
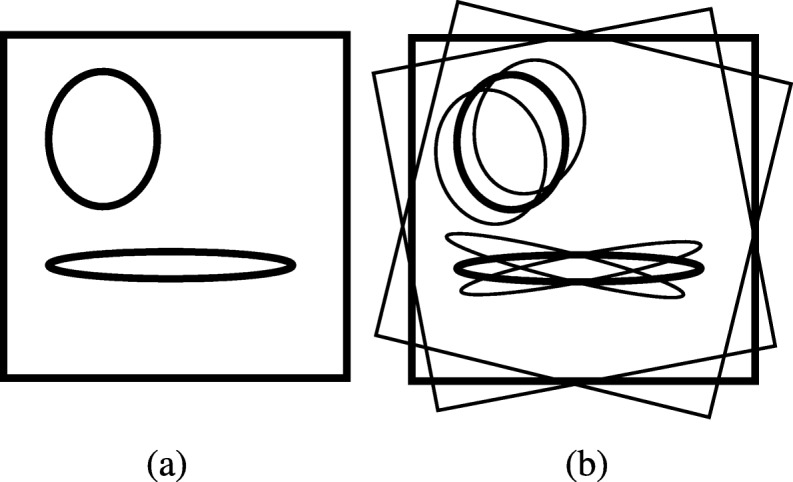
Outline diagrams for images reconstructed from **a** the original under-sampled sinogram and **b** linearly interpolated sinogram

The ultimate effect of this interpolation scheme after image reconstruction is exaggeratingly illustrated as an outline drawing in Fig. 1, where the under-sampling streaking artifacts are not shown. Figure 1a shows the main image reconstructed from the original sinogram, while Fig. 1b shows the image reconstructed from the linearly interpolated sinogram using Formula (1). It is interesting to observe from Fig. 1b that the reconstructed image from the linearly interpolated sinogram is a combination of three components: the main reconstruction using the original under-sampled sinogram (with a weighting factor of 1), a rotated version of the main reconstruction by Δγ (with a weighting factor of 0.5), and a rotated version of the main reconstruction by -Δγ (with a weighting factor of 0.5). Here 2Δγ is the angular gap between two adjacent views in the original under-sampled sinogram. In general, sinogram interpolation via linear convolution yields an image that is a combination of the main reconstruction and some rotated versions of the main reconstruction. Similar phenomena are expected for other convolution based sinogram estimation methods. The rotational artifacts are severer at locations farther away from the center of rotation.

### Estimation of un-measured data via displacement function interpolation

We believe that in order to significantly improve the sinogram estimation, we must use some sort of nonlinearity. The strategy of non-rigid deformation can be modified for our sinogram estimation task. There are many image deformation methods [19–21]. However, these methods cannot be directly applied to our sinogram estimation. One nonlinear deformation approach is sinewave fitting, which requires singular value decomposition and is rather complicated to implement [22].

The main idea of our algorithm is sketched below. A pair of measured sinogram views is provided: *p*(*n, m*_1_) and *p*(*n, m*_2_), where *n* is the index along the radial direction and *m*_1_ and *m*_2_ are two angular indices. The goal is to estimate *p*(*n, m*) with *m* between *m*_1_ and *m*_2_.

The first step of our proposed method is to find a displacement function *u*(*n*) to connect *p*(*n, m*_1_) and *p*(*n, m*_2_) so that

2 $$ p\left(n,{m}_2\right)\approx p\left(n+{u}_{m_1}^{m_2}(n),{m}_1\right) $$

We can find the function ${u}_{m_1}^{m_2}(n)$ by minimizing an objective function *F*, for given *m*_1_, *m*_2_, and *n*,

3 $$ F={\left[p\left(n,{m}_2\right)-p\left(n+{u}_{m_1}^{m_2}(n),{m}_1\right)\right]}^2+\lambda R\left({u}_{m_1}^{m_2}(n)\right) $$

with

$$ {u}_{m_1}^{m_2}(n)\in \mathbb{Z} $$

and

$$ \left|{u}_{m_1}^{m_2}(n)\right|\le N $$

where ℤ is the set of all integers and *N* is a pre-selected small positive integer. For example, *N* = 12. Here, λ is a pre-set parameter to balance the weighting between constraints in the objective function *F*. We set λ = 0.001 in our implementation of Formula (3). In Formula (3), *R* is a regularization function. If we prefer that both sides of Formula (2) have the similar trends of slopes (i.e., upward trends or downward trends), the regularization function *R* can be defined as

4 $$ R={\left[\operatorname{sign}\left(p\left(n,{m}_2\right)-p\left(n-1,{m}_2\right)\right)-\operatorname{sign}\left(p\left(n+{u}_{m_1}^{m_2}(n),{m}_1\right)-p\left(n+{u}_{m_1}^{m_2}(n)-1,{m}_1\right)\right)\right]}^2 $$

The objective function of the optimization problem is given in Formula (4), which contains two terms. The first term enforces the function displacement, which is defined in Formula (2) and can be understood as follows. We have two functions: one is labeled as *m*_1_ and the other is labeled by *m*_2_. We assume that the second function is the result of deformation from the first function. For any function value in the second function, we can find a corresponding function value in the first function. However, their associated variables differ by *u*(*n*). The second term in the objective function Formula (3) is the constraint term. The constraint is defined in Formula (4), which enforces that the slopes at the corresponding points of the two function have the same sign. In other words, if the second function at one point is increasing (or decreasing), then at the corresponding point of the first function is also increasing (or decreasing).

Normally, an objective function such as *F* in Formula (3) is minimized by an iterative gradient decent algorithm. However, if we restrict *u*(*n*) to be integers in [−*N*, *N*] with *N* being a pre-set positive integer, it is faster to evaluate the objection *F* with all possible *u*(*n*) values in [−*N*, *N*] and use a ‘*min*’ function to determine the optimal displacement function *u*(*n*). Here, ‘*min*’ is a built-in function in Matlab® to find the minimum value in an array.

The motivation of using a limited range [−*N*, *N*] is to convert an iterative optimization procedure into a one-step procedure. The selection of the integer *N* is empirical. The computation complexity is directly proportional to 2 *N* + 1. A small *N* is desirable from computation cost point of view. However, if *N* is too small, the true displacement value may be outside the range of [−*N*, *N*]. The value of *N* can be selected according to the data missing gap. If *N* were chosen to be 1000, the computation complexity is approximate that of an iterative algorithm with 2001 iterations. In order to obtain an efficient algorithm, the value of *N* must be small enough. The selection of *N* = 12 is empirical and data dependent. For different applications or different data sets this value may vary.

After the displacement function *u*(*n*) is found, in the second step, the un-measured sinogram *p*(*n, m*) with index *m* between *m*_1_ and *m*_2_ can be readily obtained by linearly interpolating the displacement function *u*(*n*). For example, if *m*_2_ – *m*_1_ = *M* + 1, we can estimate *M* views between *m*_1_ and *m*_2_ as

5 $$ p\left(n,m\right)\approx p\left(n+\frac{m-{m}_1}{M}u(n),{m}_1\right) $$

for

$$ m={m}_1,{m}_1+1,\dots, {m}_2-1. $$

We must point out in Formula (5) that *n* + *u*(*n*) × (*m* − *m*_1_)/*M* is most likely not an integer. Let

6 $$ {n}_1=n+\left\lfloor \frac{m-{m}_1}{M}u(n)\right\rfloor $$

and

7 $$ a=\left\lfloor \frac{m-{m}_1}{M}u(n)\right\rfloor -{n}_1 $$

where ⌊*x*⌋ is the largest integer that is not greater than *x*. Then Formula (5) can be implemented as the linear interpolation between two neighboring points as

8 $$ p\left(n,m\right)\approx \left(1-\alpha \right)p\left({n}_1,{m}_1\right)+\alpha p\left({n}_1+1,{m}_1\right) $$

for

$$ m={m}_1,{m}_1+1,\dots, {m}_2-1. $$

### Image reconstruction

The k-space data is complex in nature. Our proposed sinogram estimation method described in Section "Estimation of un-measured data via displacement function interpolation" was developed for real functions. The 1D inverse Fourier transform for the radial k-space measurements is first performed view-by-view. The result is the spatial-domain sinogram. This spatial-domain sinogram has a real part and an imaginary part. The sinogram extension method described in Section "Estimation of un-measured data via displacement function interpolation" is applied to the magnitude of the sinogram. The image reconstruction algorithm is chosen as the FBP algorithm. This FBP algorithm in Matlab® is a built-in function ‘*iradon*’.

In computer simulations, we demonstrate our method with a real-valued (magnitude) sinogram. The original under-sampled sinogram was generated analytically without noise. We performed three computer simulation studies and one patient study. In the first computer simulation study, the original measured number of views was 60 over 360°. After sinogram extension, the number of views was increased to 180 over 360°. In the second computer simulation study, the original measured number of views was 120 over 360°. After sinogram extension, the number of views was increased to 360 over 360°. The absolute error image between the estimated sinogram and the true sinogram was calculated and reported in the next section.

For the patient cardiac perfusion MRI study, a Siemens 3 T Trio scanner was used for data acquisition [23]. We used a phased array of coils, one of which was chosen to demonstrate the proposed method. The scanner parameters for the radial acquisition were TR = 2.6 ms, TE = 1.1 ms, flip angle = 12°, Gd dose = 0.03 mmol/kg, and slice thickness = 6 mm. Reconstruction pixel size was 1.8 × 1.8 mm^2^. Each image was acquired in a 62 ms readout. The acquisition matrix size for an image frame was 256 × 72, and 75 sequential images were obtained at 75 different times. At each time frame, the k-space is sampled with 72 uniformly spaced radial lines over an angular range of 180°.

To illustrate our proposed algorithm, at each time frame we under-sampled the 72 views into 24 views for image reconstruction. The images with 72 views were treated as the gold standard. Only one timeframe was used at a time.

For the patient study, the state-of-the-art iterative total variation (TV) algorithm was also used for image reconstruction with under-sampled MRI data. The number of iterations was 1000. The reconstructions were compared with the gold standard images obtained with 72 views in terms of root mean square error (RMSE).

## Results

### Computer simulations

**Fig. 2:**
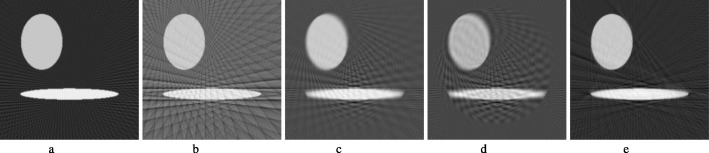
FBP reconstructions reconstructed by **a** 180 measured views, **b** 60 measured views, **c** 180 views created from 60 views by linear interpolation, **d** 180 views created from 60 views by sinc function interpolation, and **e** 180 views created from 60 views by proposed method

Figure 2 shows the results from the computer simulations with the FBP reconstruction algorithm. In this figure, measurements from 180 views over 360° are considered as a full sinogram, and measurements from 60 views over 360° are considered as an under-sampled sinogram. Figure 2a and b show the FBP reconstruction results from the full and under-sampled sinograms, respectively. Figure 2c and d show the results with linear convolution sinogram interpolation methods: linear interpolation and sinc function (convolution) interpolation. The linear interpolation method is equivalent to the triangle function (convolution) interpolation method. Figure 2e shows the result of the proposed non-linear method.

**Fig. 3:**
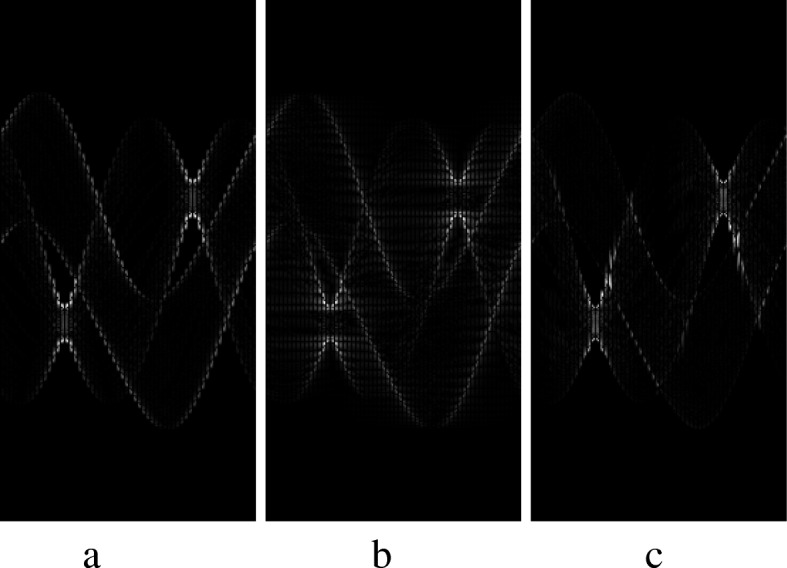
Absolute error between the estimated sinogram and the true sinogram of 180 views, using **a** linear interpolation, **b** sinc function interpolation, and **c** proposed displacement function interpolation method

The estimated sinograms and the true sinogram are compared in terms of the absolute value of the difference in Fig. 3 for the estimation methods used in Fig. 2. A summary of the absolute errors is listed in Table 1.

**Table 1:** Computer simulation set #1 sinogram estimation errors

Methods	Maximal absolute error	Sum of absolute errors
Linear interpolation	0.1676	193.0636
Sinc interpolation	0.1762	128.5682
Proposed	0.1439	87.6291

### Patient study

**Fig. 4:**
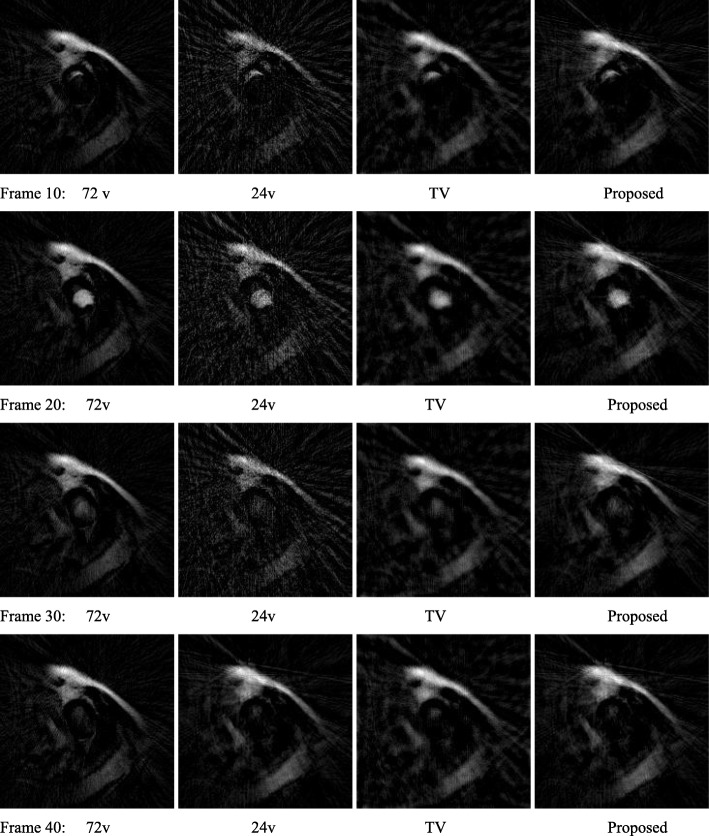
(Frame 10 ~ 40). MRI patient image reconstructions by using (1st column) 72-view FBP, (2nd column) 24-view FBP, (3rd column) 24-view iterative TV, and (4th column) 24-view proposed method. The 1st column is treated as the gold standard for other columns to compare with. The four rows of images correspond to time frames of 10, 20, 30, and 40, respectively

**Fig. 5:**
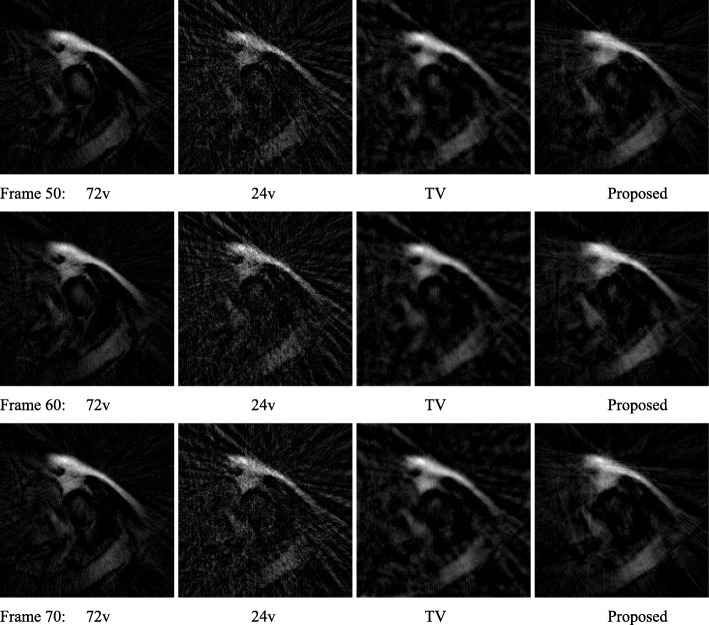
(Frame 50 ~ 70). MRI patient image reconstructions by using (1st column) 72-view FBP, (2nd column) 24-view FBP, (3rd column) 24-view iterative TV, and (4th column) 24-view proposed method. The 1st column is treated as the gold standard for other columns to compare with. The three rows of images correspond to time frames of 50, 60 and 70, respectively

A de-identified MRI data set was used for a comparison study. Both state-of-the-art iterative TV algorithm and proposed non-iterative algorithm were used to reconstruct the images. The results of the patient cardiac perfusion MRI study are shown in figures radial data. Figures 4 and 5’s second columns show the FBP reconstruction using the under-sampled 24-view data. Figures 4 and 5’s third columns show iterative TV reconstruction using the under-sampled 24-view data. Figures 4 and 5’s forth columns show the FBP reconstruction using the extended data by the proposed displacement function method from the 24-view data. The values of *u*(*n*) was restricted to be integers in the range of [− 12, 12].

The state-of-the-art iterative TV algorithm provides the least noisy images. However, according to the RMSE analysis shown in Table 2, the proposed non-iterative method has the smallest error compared to the gold standard, which uses 72 views.

**Table 2:** RMSE for various reconstruction methods

	72 views FBP	24 views FBP	24 views Iterative TV	24 views Proposed
Time frame 10	0	0.0907	0.0717	0.0647
Time frame 20	0	0.0996	0.0704	0.0661
Time frame 30	0	0.0893	0.0690	0.0631
Time frame 40	0	0.0986	0.0720	0.0623
Time frame 50	0	0.0893	0.0693	0.0592
Time frame 60	0	0.0905	0.0633	0.0615
Time frame 70	0	0.0932	0.0699	0.0581

## Discussion and conclusions

This paper observes that linear convolution based sinogram interpolation method may produce rotation artifacts. To overcome this problem, we propose a nonlinear method that relates the adjacent measurements by a displacement function, and the linear interpolation of the displacement function yields the estimation of un-measured data.

In this proposed method, two adjacent measured views in the original under-sampled sinogram are used for missing data estimation. A displacement function, *u*(*n*), is estimated by minimizing an objective function, *F*. We restrict the values of the displacement function *u*(*n*) to be integers in a small range [−*N*, *N*], say, *N* = 12. The minimization procedure can be non-iterative. We use the ‘*min*’ function to find the optimal solution for each index *n*. Then linear interpolation of *u*(*n*) is performed for each un-measured view between the two adjacent measured views. For example, if the un-measured view is exactly at the middle between the two measured views, the interpolated displacement function for this un-measured view is 0.5 × *u*(n). Most likely 0.5 × *u*(n) is not an integer. Linear interpolation is required to form the estimated sinogram *p*(*n* + 0.5*u*(*n*), *m*). Finally, the image is reconstructed by the FBP algorithm, in which the k-space re-gridding is not required.

One advantage of the proposed method is that the resultant FBP reconstruction using the estimated sinogram does not have the rotation artifacts. Our estimated sinogram is more accurate than the sinogram estimated by linear-convolution-based methods. This point is demonstrated by the absolute errors as shown in Table 1. Thanks to the FBP algorithm, the proposed method does not suffer from the k-space re-gridding errors.

In our patient study, there are 24 views over 180°. This number of views is extremely small, much smaller than the recommended view numbers in clinical applications. The most significant feature of our algorithm is that no prior information is ever assumed in the proposed method. Our proposed algorithm is compared against the state-of-the-art iterative TV algorithm using the patient dynamic MRI data set. The iterative TV algorithm provides less-noisy images. However, the proposed non-iterative algorithm produces the images that have less RMSE errors when compared with the 72-view gold standard images. The unique characteristic of the proposed algorithm is its non-iterative nature and efficient computation.

The main motivation for us to use the displacement function method is that the displacement function method is nonlinear, because we observe that linear methods cause rotational artifacts in the image. Other nonlinear methods may also work to estimate the missing data.

## Data Availability

Not applicable.

## Abbreviations

1D: One dimensional
FBP: Filtered backprojection
MRI: Magnetic resonance imaging
RMSE: Root mean square error
TV: Total variation
